# Limb remote ischemic preconditioning attenuates biomarkers of acute lung injury and inflammation during thoracoscopic lobectomy: a randomized controlled trial

**DOI:** 10.1016/j.clinsp.2026.100883

**Published:** 2026-02-17

**Authors:** Wenfu Zhang, Mingwang Zeng, Chao Yang, Lijun Yang, Juan Yang, Yi Wang, Haiyu Xie, Lifeng Wang, Maolin Zhong, Fuzhou Hua, Weidong Liang

**Affiliations:** a Anesthesia & Surgery Center, The First Affiliated Hospital of Gannan Medical University, Jiangxi, People's Republic of China; b Gannan Medical University, Jiangxi, People's Republic of China; c Hospital of Traditional Chinese Medicine of Zhongshan, Zhongshan, Guangdong, People's Republic of China; d Fujian Maternity and Child Health Hospital College of Clinical Medicine for Obstetrics & Gynecology and Pediatrics, Fujian Medical University, Fuzhou, People's Republic of China; e Academic Affairs Department, The First Affiliated Hospital of Gannan Medical University, Jiangxi, People's Republic of China; f Department of Anesthesiology, The Second Affiliated Hospital of Nanchang University, Jiangxi, People's Republic of China

**Keywords:** Remote ischemic preconditioning, One-lung ventilation, Acute lung injury, Thoracoscopic surgery, Randomized controlled trial

## Abstract

**Background:**

One-Lung Ventilation (OLV), though essential for thoracic surgery, triggers inflammation and oxidative stress that may lead to Acute Lung Injury (ALI). Remote Ischemic Preconditioning (RIPC) is a simple, non-invasive intervention that may activate protective pathways and reduce ischemia-reperfusion injury. This randomized controlled trial assessed whether limb RIPC attenuates OLV-induced lung injury during thoracoscopic lobectomy.

**Methods:**

Fifty-four patients undergoing thoracoscopic lobectomy with OLV were randomized to the RIPC (*n* = 27) or Control (NC, *n* = 27) group. RIPC consisted of five cycles of 5-min ischemia and 5-min reperfusion using a lower limb tourniquet before OLV. Blood samples were collected at T0 (post-induction), T1 (30-min after OLV), T2 (90-min after OLV), and T3 (30-min after resumption two-lung ventilation). The primary endpoint was plasma CC16. Secondary endpoints included IL-6, MDA, arterial blood gases, Intraoperative Oxygenation Indices (OI, RI, A-aDO_2_, a/A), and hospital stay.

**Results:**

Plasma CC16, IL-6, and MDA levels were significantly lower in the RIPC group from T1 to T3 (all *p* < 0.001). In contrast, oxygenation indices, blood gas parameters, and hospital stay did not differ significantly between groups (all *p* > 0.05).

**Conclusions:**

Limb RIPC effectively reduced systemic markers of lung injury (CC16), inflammation and oxidative stress during OLV but not in short-term improvements in oxygenation or clinical recovery. These findings suggest that RIPC confers molecular protection, with potential functional benefits requiring longer follow-up or evaluation in high-risk populations. Larger multicenter trials are warranted.

## Highlights

• Limb RIPC decreases plasma CC16, a key biomarker of lung epithelial injury.
• RIPC attenuates systemic Inflammation (IL-6) and oxidative stress (MDA).
• A simple, non-invasive intervention for molecular lung protection.
• No short-term improvement in oxygenation indices was observed postoperatively.

## Introduction

Lung cancer remains a leading cause of cancer-related mortality worldwide. According to global epidemiological data, the incidence of lung cancer continues to rise, with an estimated 2 million new cases and 1.76 million deaths annually.^1^ Pulmonary lobectomy is the primary curative treatment for early-stage lung cancer, yet it carries a risk of serious postoperative complications, among which Acute Lung Injury (ALI) is one of the most severe. The incidence of ALI following pulmonary resection is reported to range from 2 % to 8 %, with a subset of cases progressing to Acute Respiratory Distress Syndrome (ARDS), respiratory failure, Multiple Organ Dysfunction Syndrome (MODS), or even mortality.^2^ A major contributor to ALI in thoracic surgery is One-Lung Ventilation (OLV),^3^ which is routinely employed during pulmonary lobectomy to optimize surgical exposure and prevent cross-contamination between lungs. However, OLV induces substantial lung stress, leading to Ischemia-Reperfusion (I/R) injury, excessive oxidative stress, and an exaggerated inflammatory response. The ventilated lung experiences volutrauma and hyperoxia-induced injury, while the non-ventilated lung suffers from hypoxia-reoxygenation stress upon re-expansion. Together, these pathological processes elevate inflammatory cytokine release, promote oxidative damage, and increase alveolar-capillary permeability, all of which contribute to postoperative ALI.^4^

Given the impact of ALI on surgical outcomes, perioperative lung protection strategies have garnered increasing attention in anesthetic and surgical management. Current lung protective approaches include pharmacologic interventions ‒ such as sevoflurane preconditioning, dexmedetomidine, glucocorticoids, protease inhibitors, and propofol ‒ as well as ventilation strategy modifications, including low tidal volume ventilation, Positive End-Expiratory Pressure (PEEP), and permissive hypercapnia.^5^ While these interventions provide varying degrees of lung protection, their effectiveness remains limited, necessitating novel, non-invasive strategies to mitigate OLV-induced lung injury and improve postoperative pulmonary function.

Limb Remote Ischemic Preconditioning (RIPC) has emerged as a promising organ-protective technique against I/R injury. RIPC involves repeated, transient, and non-lethal ischemia-reperfusion cycles applied to a distant limb, triggering endogenous protective mechanisms that enhance the target organ’s resilience to ischemic and oxidative stress.^6^^,^^7^ Evidence suggests that RIPC confers multi-system protection, including cardioprotection, neuroprotection, nephroprotection, and hepatoprotection, by attenuating endothelial dysfunction, reducing neutrophil activation, suppressing systemic inflammation, and limiting oxidative stress.^8^, ^9^, ^10^, ^11^, ^12^, ^13^ Although RIPC has demonstrated protective effects on the heart, kidney, brain, and liver, its potential role in lung protection remains underexplored. Few studies have investigated whether RIPC can directly attenuate lung injury in patients undergoing pulmonary resection, and the underlying mechanisms remain unclear. Given its non-invasive nature, ease of application, and potential broad clinical benefits, RIPC represents a compelling candidate for perioperative lung protection.

This study aimed to investigate whether limb RIPC could mitigate lung injury and improve oxygenation parameters in patients undergoing thoracoscopic-assisted pulmonary lobectomy. By evaluating its lung-protective effects and potential mechanisms, the authors seek to provide novel insights into perioperative lung protection strategies and expand the clinical applicability of RIPC in thoracic surgery.

## Materials and methods

### Study design and participants

This study was designed as a prospective, single-center, randomized, single-blind clinical trial conducted at the First Affiliated Hospital of Gannan Medical University. The trial was approved by the Research Ethics Committee (Approval n° LLSC-2021071901, date of approval: July 13, 2021) and complied with the principles of the Helsinki Declaration and the CONSORT guidelines. The trial was registered in the Chinese Clinical Trial Registry (ChiCTR2100049712, date of registration: August 8, 2021). Written informed consent was obtained from all participants after a thorough explanation of the study protocol.

From November 2021 to July 2022, a total of 54 lung cancer patients undergoing elective thoracoscopic lobectomy were recruited. Randomization was conducted using a computer-generated sequence by an independent investigator, with participants randomly assigned (1:1) to either the RIPC group or the NC (non-conditioning) group.

### Inclusion criteria

Eligible patients met the following criteria:

1. Age between 30 and 80-years.
2. American Society of Anesthesiologists (ASA) classification I‒II.
3. Normal cognitive function, no surgical contraindications.
4. No history of long-term use of antioxidants (e.g., multivitamins), corticosteroids, or Nonsteroidal Anti-Inflammatory Drugs (NSAIDs).
5. Complete clinical data with signed informed consent from patients and their families.

### Exclusion criteria

Patients were excluded if they had:

1. Moderate-to-severe anemia or hypoproteinemia.
2. Severe cardiovascular or renal dysfunction, including: a) Uncontrolled hypertension (> 160/100 mmHg). b) Diabetes mellitus with complications. c) Coronary artery disease.
3. Preoperative severe respiratory dysfunction, defined as: a) Arterial Oxygen Pressure (PaO₂) < 60 mmHg b) Forced Expiratory Volume in 1-second (FEV₁) < 50 % of predicted value.
4. Coagulation disorders and/or thrombocytopenia.
5. History of preoperative chemotherapy or radiotherapy.
6. Active infection, indicated by body temperature > 38 °C or elevated C-Reactive Protein (CRP).
7. History of asthma.
8. Peripheral vascular disease, muscle disorders, or limb thrombosis.

### Intraoperative management and protocol deviations

Patients were excluded from the study if they experienced:

1. If intraoperative conversion to thoracotomy occurred, the surgical procedure no longer met the predefined inclusion criterion (thoracoscopic lobectomy). These cases were considered protocol deviations and were not included in the per-protocol analysis. However, all randomized patients were retained in the intention-to-treat analysis.

### Remote ischemic preconditioning (RIPC) protocol

The limb RIPC protocol was applied after anesthesia induction and before surgery. In the RIPC group, an inflatable limb ischemic preconditioning device (Shenzhen, model RIP-809S) was applied to the thigh on the non-operative side, positioned 4–5 cm above the knee joint. The cuff was inflated to 200 mmHg for 5 cycles, each consisting of 5 minutes of ischemia (inflation), followed by 5 minutes of reperfusion (deflation). In the NC group, an identical device was applied without inflation to maintain blinding.

### Anesthesia management

Perioperative monitoring and induction

Upon arrival in the operating room, patients underwent standard monitoring, including: Heart Rate (HR), Noninvasive Blood Pressure (NIBP), Peripheral Oxygen Saturation (SpO_2_), and Electrocardiography (ECG). Invasive Blood Pressure (IBP) via radial artery catheterization. A peripheral venous catheter was inserted for fluid and drug administration. Anesthesia was induced using Midazolam (0.04 mg/kg, IV), Propofol (2.5–3 mg/kg, IV), Sufentanil (0.4–0.5 μg/kg, IV), and Rocuronium bromide (0.6–0.9 mg/kg, IV) for neuromuscular blockade. After loss of consciousness, a Double-Lumen endotracheal Tube (DLT) was inserted and confirmed using bronchoscopy.

### Ventilation strategy

During Two-Lung Ventilation (TLV): Tidal Volume (VT): 8 mL/kg, Respiratory rate (f): 12 breaths/min, I:E ratio: 1:2. During One-Lung Ventilation (OLV): VT: 6 mL/kg, f: 14–16 breaths/min, Fraction of Inspired Oxygen (FiO_2_): 100 % Oxygen flow: 2 L/min, End-expiratory CO_2_: maintained at 35–45 mmHg. After confirming tube positioning, a right internal jugular vein catheter was inserted under ultrasound guidance.

### Anesthesia maintenance

Anesthesia was maintained with: Propofol (4–8 mg/kg/h, IV infusion). Remifentanil (0.1–0.2 μg/kg/min, IV infusion). Sevoflurane (0.8 %–1.5 %) (maintaining BIS 40–60). Intermittent rocuronium (0.1–0.2 mg/kg, IV) as needed for muscle relaxation. At the end of surgery, ventilation was switched back to TLV, and patients received: Sufentanil (5–10 μg, IV) for analgesia Transfer to the Post-Anesthesia Care Unit (PACU) with FiO_2_ 0.5 L/min. Postoperative analgesia was managed via patient-controlled IV infusion pump containing: Sufentanil (80–100 μg), Ondansetron (8 mg), Normal saline (100 mL), (Infusion rate: 2 mL/h, lockout time: 10 min)

### Outcomes

#### Primary outcome

Serum Clara Cell secreted protein (CC16), a biomarker of lung epithelial injury.

Secondary outcomes: Inflammatory Cytokines (IL-6), serum Malondialdehyde (MDA). Oxygenation parameters: Oxygenation Index (OI = PaO_2_/FiO_2_), Respiratory Index (RI), Alveolar-arterial Oxygen gradient (A-aDO_2_), arterial-alveolar oxygen ratio (a/A ratio), pH levels.

### Blood sample collection and biomarker sub-sample analysis

Arterial blood samples were drawn at four time points: T0: After anesthesia induction, T1: 30-min after OLV initiation, T2: 90-min after OLV initiation, and T3: 30-min after the resumption of TLV. pH, blood Partial Oxygen pressure (PaO_2_), blood partial Carbon Dioxide pressure (PaCO_2_) and Lactate (Lac) concentrations were measured. According to the formula, the OI, RI, A-aDO_2_ and a/A ratio were calculated. Two milliliters of venous blood were collected from the internal jugular vein at the above four time points and centrifuged at 3000 rpm for 15 min, and the plasma was stored at −80 °C. According to the literature,^14^ the authors randomly selected 20 blood samples from the NC group and 20 blood samples from the RIPC group. Serum CC16, IL-6 and MDA concentrations were determined by enzyme-linked immunosorbent assay (rB, Atlanta, USA). Postoperative extubating time, PACU duration, and hospital stay were also recorded.

### Sample size and blinding

The sample size was calculated based on the primary endpoint, the Respiratory Index (RI), according to the relevant literature. Assuming a mean difference of 0.23 between the two groups with the standard deviation of 0.28, a two-sided α of 0.05, and a power (1-β) of 0.8. Twenty-four patients were required in each group. To account for an estimated 10 % dropout rate, 27 patients were finally enrolled per group.

This was a single-blind study. Patients were blinded to group allocation throughout the trial. Investigators responsible for intraoperative data collection, postoperative outcome assessment, and laboratory analyses of blood samples (biomarker assays) were also blinded to group assignment. The anesthesiologists who performed the randomization and implemented the intervention were not involved in data collection or analysis.

### Statistical analysis

Descriptive statistics were applied to summarize baseline characteristics. Continuous variables are presented as mean ± Standard Deviation (SD), and categorical variables as frequencies and percentages. Between-group comparisons of categorical variables were conducted using the χ^2^ test or Fisher’s exact test, as appropriate. Continuous outcomes, including plasma biochemical markers measured at multiple time points, were analyzed with repeated-measures analysis of variance (ANOVA) to evaluate both within-group changes over time and between-group differences. Missing data were handled using listwise deletion. Sphericity was evaluated using Mauchly’s test, and the Greenhouse-Geisser correction was applied when the assumption of sphericity was violated. All statistical analyses were performed using SPSS version 26.0 (IBM, Armonk, NY, USA). All statistical tests were two-tailed, and a p-value < 0.05 was considered statistically significant.

## Results

### Baseline and intraoperative characteristics

**Fig. 1:**
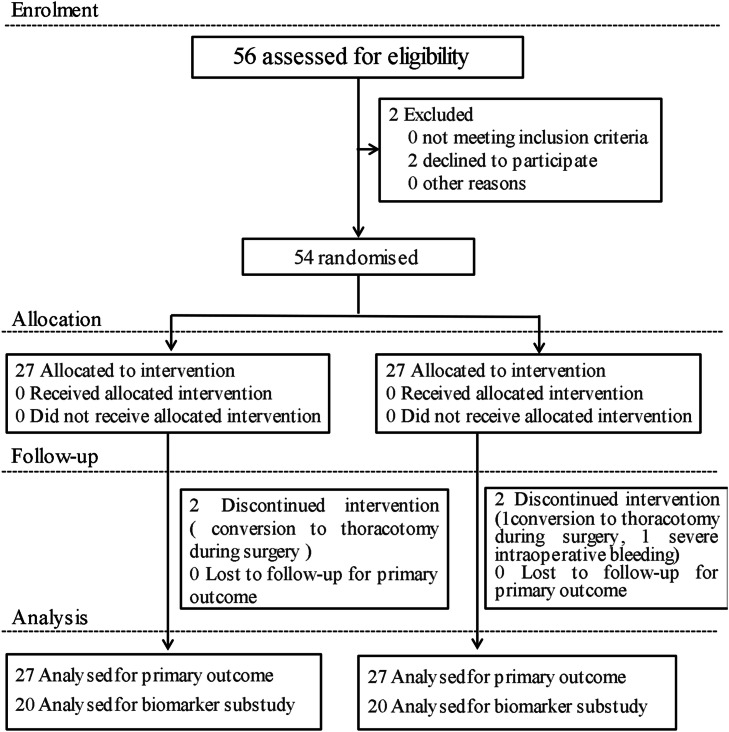
CONSORT flow diagram illustrating participant recruitment, allocation, follow-up, and analysis in the randomized clinical trial.

A total of 54 patients were initially enrolled between November 2021 and July 2022. Four patients were excluded: three due to conversion to thoracotomy and one due to severe intraoperative bleeding. Thus, 54 patients were ultimately included in the final Intention-to-treat analysis. As predefined in the study protocol, biomarker analysis was conducted in a randomly selected subset of 40 patients (20 per group). The study flow diagram is presented in Fig. 1.

Baseline demographic and intraoperative characteristics of patients are summarized in Table 1. There were no statistically significant differences between the RIPC and NC groups in terms of age, sex, Body Mass Index (BMI), anesthesia duration, One-Lung Ventilation (OLV) duration, operative time, urine output, duration of PACU stay, or total length of hospitalization (*p* > 0.05).

**Table 1:** Baseline demographic and perioperative characteristics of patients.

**Characteristic**	**Control group** **(*n* = 25)**	**RIPC group** **(*n* = 25)**
Age (years), mean ± SD	60.4 ± 8.54	58.28 ± 11.45
BMI (kg/m^2^), mean ± SD	23.22 ± 2.21	23.62 ± 3.19
Sex, males, *n* (%)	11 (44 %)	10 (40 %)
Left-side surgery, *n* (%)	7 (28 %)	9 (36 %)
Operation time (min), mean ± SD	160.6 ± 40.27	163.8 ± 56.22
Intraoperative fluid load (mL), mean ± SD	1492 ± 232.9	1702 ± 502.6
Estimated blood loss (mL), mean ± SD	156 ± 69.7	184 ± 98.66
Urine output (mL), mean ± SD	738.4 ± 201.43	818 ± 189.78
Duration of anesthesia (min), mean ± SD	204.4 ± 37.29	219.6 ± 59.56
OLV duration (min), mean ± SD	142.6 ± 35.77	154.4 ± 45.79
Length of PACU stay (min), mean ± SD	90.2 ± 36.01	91 ± 36.14
Length of hospital stay(days), mean ± SD	8 ± 1.61	8 ± 1.9

BMI, Body Mass Index; PACU, Post-Anesthesia Care Unit; RIPC, Remote Ischemic Preconditioning; OLV, One-Lung Ventilation.

### Primary outcome: lung injury biomarker (CC16)

**Fig. 2:**
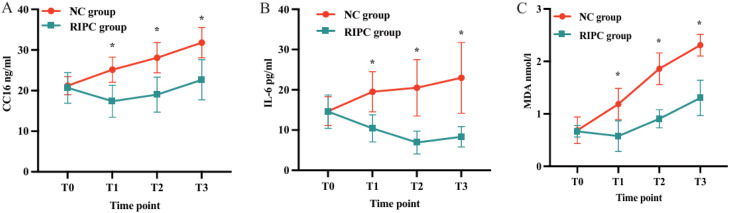
Time-course comparison of plasma biomarkers between the Remote Ischemic Preconditioning (RIPC) group and the Non-Conditioning Control (NC) group: (A) Clara Cell secreted protein 16 (CC16), (B) Interleukin-6 (IL-6), (C) Malondialdehyde (MDA). Data are presented as mean ± SD. *p < 0.001 vs. NC group at the corresponding time point.

As shown in Fig. 2A, plasma levels of Clara Cell secretory protein (CC16), a serological marker of lung epithelial injury, were significantly lower in the RIPC group compared with the NC group at time points T1, T2, and T3 (*p* < 0.001). The trend over time showed a significant group-time interaction, indicating that RIPC was associated with reduced CC16 release following OLV-induced lung stress.

### Secondary outcomes: inflammatory and oxidative stress markers

Plasma levels of Malondialdehyde (MDA) and Interleukin-6 (IL-6) were also significantly lower in the RIPC group at T1–T3 compared to the NC group (Fig. 2B and C, all *p* < 0.001). These findings suggest that RIPC effectively attenuated systemic oxidative stress and inflammatory responses associated with OLV.

### Oxygenation and gas exchange parameters

**Fig. 3:**
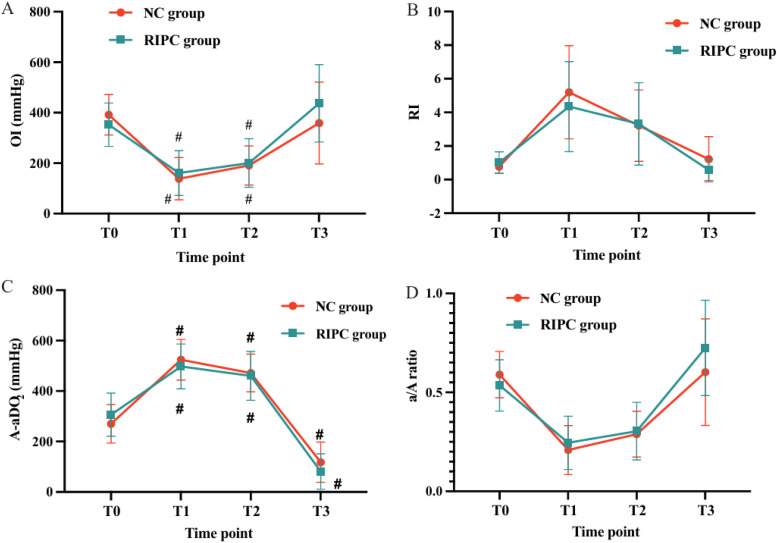
Comparison of respiratory parameters over time between the RIPC and NC groups: (A) Oxygenation Index (OI), *p* = 0.396, (B) Respiratory Index (RI), *p* = 0.423, (C) Alveolar-arterial Oxygen gradient (A-aDO_2_), *p* = 0.559, (D) Arterial-alveolar oxygen ratio (a/A ratio), *p* = 0.347. Data are presented as mean ± SD. No statistically significant differences were observed between groups.

While no statistically significant differences were observed in Oxygenation Index (OI), Respiratory Index (RI), Alveolar-arterial Oxygen Difference (A-aDO_2_), or arterial-alveolar oxygen ratio (a/A ratio) between the two groups (all *p* > 0.05), overall gas exchange parameters remained comparable throughout the study period (Fig. 3D, Table 2).

**Table 2:** Blood gas parameters and lung function variables at different time points in the RIPC and control groups.

**Variable**	**Group**	**T0 (Baseline)**	**T1 (30-min after OLV)**	**T2 (90-min after OLV)**	**T3 (30-min after DLV)**
**Arterial PH**	Control group	7.41±0.06	7.40±0.06	7.39±0.06	7.30±0.05^a^
	RIPC group	7.39±0.05	7.37±0.05	7.36±0.05	7.31±0.07^a^
**PaCO_2_**	Control group	41.00±6.30	40.12±6.65	40.40±5.83	47.24±8.50
	RIPC group	43.48±6.63	43.12±5.42	41.88±5.81	45.55±9.50
**Lac**	Control group	0.86±0.33	0.75±0.31	0.70±0.22	0.76±0.28
	RIPC group	0.91±0.57	0.78±0.43	0.71±0.30	0.83±0.42
**a/A ratio**	Control group	0.59±0.12	0.21±0.12^a^	0.29±0.12^a^	0.60±0.27
	RIPC group	0.54±0.13	0.25±0.13^a^	0.30±0.15^a^	0.72±0.24
**A-aDO_2_**	Control group	270.11±76.67	524.25±80.76^a^	471.70±74.99^a^	117.89±79.51^a^
	RIPC group	306.37±85.40	497.82±89.09^a^	460.17±96.50^a^	81.18±69.91^a^
**RI**	Control group	0.76±0.38	5.20±2.77^a^	3.21±2.13^a^	1.21±1.35
	RIPC group	1.01±0.64	4.34±2.68^a^	3.32±2.45^a^	0.57±0.53
**OI**	Control group	391.64±79.95	138.6 ± 83.54^a^	190.8 ± 77.36^a^	359.12±16.29
	RIPC group	352.28±86.22	161.28±88.68^a^	200.48±96.37^a^	437.04±153.3

Values are presented as means ± SD. OLV, One-Lung Ventilation; DLV, Double-Lung Ventilation; PaCO₂, Arterial Partial pressure of Carbon Dioxide; PaO_2_, Arterial Partial Pressure of Oxygen; a/A ratio, Arterial-to-Alveolar Oxygen tension ratio; A-aDO_2_, Alveolar-to-Arterial Oxygen tension gradient; RI, Respiratory Index; OI, Oxygenation Index, RIPC, Remote Ischemic Preconditioning.

a *p* < 0.05 compared with baseline (T0).

### Hemodynamic and respiratory mechanics

There were no significant differences in heart rate, mean arterial pressure, plateau pressure, or peak airway pressure between the two groups at any time point (Table 3, all *p* > 0.05), indicating that RIPC did not adversely affect intraoperative hemodynamics or mechanical ventilation parameters.

**Table 3:** Hemodynamic and respiratory pressure parameters at different time points in the RIPC and control groups.

**Variable**	**Group**	**T0 (Baseline)**	**T1 (30-min after OLV)**	**T2 (90-min after OLV)**	**T3 (30-min after DLV)**
HR (beats/min)	Control group	67.72±5.86	69.72±5.78	67.88±4.95	68.44±3.23
	RIPC group	71.96±9.00	71.72±8.16	70.96±8.23	69.88±7.45
MAP (mmHg)	Control group	81.52±14.11	79.44±14.53	88.20±11.75	86.36±10.40
	RIPC group	85.20±14.47	85.20±13.30	90.36±12.85	90.00±9.01
Ppeak (cmH_2_O)	Control group	14.96±3.13	24.12±3.64	24.88±2.77	15.80±2.45
	RIPC group	14.8 ± 2.86	25.2 ± 3.98	25.64±3.70	16.2 ± 2.60
Pplat (cmH_2_O)	Control group	13.6 ± 2.60	23.00±3.64	23.68±3.04	15.00±2.68
	RIPC group	13.52±2.90	23.64±4.06	24.04±3.58	15.36±2.97

Data are presented as mean ± SD.

HR, Heart Rate; MAP, Mean Arterial Pressure; Ppeak, Peak airway pressure; Pplat, Plateau airway pressure; OLV, One-Lung Ventilation; DLV, Double-Lung Ventilation; RIPC, Remote Ischemic Preconditioning. All *p* > 0.05 between the two groups at all time points.

### Pathophysiological insight

OLV-related lung injury is primarily driven by two main mechanisms: hypoxia-induced lung injury and mechanical ventilation-induced stretch injury.^4^ Hypoxia promotes excessive generation of Reactive Oxygen Species (ROS), leading to damage to alveolar epithelial and endothelial cells, along with the release of inflammatory mediators. Furthermore, ischemia and reperfusion injury during OLV impairs alveolar type II cell function, resulting in reduced surfactant secretion, elevated alveolar surface tension, and subsequent pulmonary edema.^15^ The present findings indicate that RIPC may attenuate these processes by reducing oxidative stress and systemic inflammation, as evidenced by the significant decreases in CC16, IL-6, and MDA levels observed in the RIPC group.

## Discussion

Remote Ischemic Preconditioning (RIPC) is increasingly recognized as a safe, feasible, and effective strategy for mitigating Ischemia-Reperfusion (IR) injury across various organ systems.^16^ RIPC involves transient and reversible ischemia-reperfusion cycles applied to a remote organ or tissue, which in turn induces systemic protective mechanisms through humoral, neural, and inflammatory pathways.^17^ During thoracoscopic pulmonary lobectomy, patients undergoing One-Lung Ventilation (OLV) are particularly vulnerable to oxidative stress and inflammatory cascades, which may disrupt pulmonary vascular endothelial and epithelial barriers, increasing alveolar-capillary permeability and contributing to Acute Lung Injury (ALI).^15^ In severe cases, this injury can escalate to Acute Respiratory Distress Syndrome (ARDS) or even death.

In this study, the authors demonstrated that preoperative RIPC significantly reduced lung epithelial injury, inflammatory cytokine release, and oxidative stress markers in patients undergoing thoracoscopic lobectomy. Specifically, the plasma levels of Clara Cell secretory protein (CC16), a biomarker of epithelial lung injury, were significantly lower in the RIPC group at all post-OLV time points (T1–T3). Similarly, RIPC effectively reduced Interleukin-6 (IL-6) and Malondialdehyde (MDA) levels, which are reliable indicators of systemic inflammation and oxidative stress, respectively. However, despite these biomarker improvements, no statistically significant differences were observed in Oxygenation Index (OI), Respiratory Index (RI), or Alveolar-Arterial Oxygen gradient (A-aDO_2_) between groups, nor in clinical outcomes such as hospital stay duration.

### CC16 as a biomarker of lung injury

CC16 is secreted by non-ciliated Clara cells in bronchioles and is considered a sensitive marker of airway epithelial barrier integrity. Acute lung injury and pulmonary irritants can lead to transient increases in plasma CC16, largely reflecting increased bronchoalveolar-capillary permeability.^18^ Elevated serum CC16 levels correlate with lung injury severity and may serve as a predictor of infection severity and clinical outcomes.^19^ In our study, RIPC was associated with consistently lower CC16 levels during and after OLV, suggesting a protective role in preserving epithelial barrier function and mitigating OLV-induced lung damage.

### Inflammatory response and IL-6

The present findings also revealed that plasma IL-6 levels were significantly lower in the RIPC group. The IL-6 increase was delayed and less pronounced, peaking only after prolonged OLV (90-min), whereas the Control group showed an immediate and marked increase. This suggests that RIPC may blunt the surgical inflammatory stress response and modulate cytokine release. Prior animal studies have similarly reported elevated IL-6 and TNF levels during lung recruitment following OLV, implicating reperfusion injury and mechanical stress in the pathogenesis of pulmonary inflammation.^20^^,^^21^ IL-6 plays a central role in acute-phase protein synthesis, leukocyte recruitment, and inflammation amplification. Its level correlates with trauma severity, operative time, and postoperative complications, making it a reliable marker of surgical stress.^22^ These results reinforce this role and indicate that RIPC reduces inflammatory load and may mitigate inflammatory lung injury.

### Oxidative stress and MDA

MDA, a lipid peroxidation byproduct, serves as a reliable indicator of oxidative stress. Prior studies show that MDA levels increase significantly with prolonged OLV and are particularly elevated following lung re-expansion.^23^ In this trial, both groups experienced rising MDA levels after OLV initiation, but the RIPC group consistently maintained lower MDA concentrations, suggesting that RIPC activates endogenous antioxidant responses and reduces ROS generation under stress.

### Respiratory indices and gas exchange

No statistically significant differences were detected in OI, RI, or A-aDO_2_ between the two groups. Both groups demonstrated comparable changes in pulmonary oxygenation and diffusion function during and after OLV (Table 2). OI, calculated as PaO_2_/FiO_2_, reflects oxygenation efficiency, whereas A-aDO_2_ represents gas exchange gradients, serving as a surrogate for lung injury severity.

Although group differences did not reach statistical significance, favorable trends were observed in the RIPC group. OI showed an upward trajectory, while A-Ado_2_ and RI declined post-OLV compared with the control group (Table 2). These findings may hint at modest improvements in pulmonary oxygenation and diffusion function. OI, calculated as PaO_2_/FiO_2_, reflects oxygenation efficiency, whereas A-aDO_2_ represents gas exchange gradients, serving as a surrogate for lung injury severity.^10^^,^^24^ Although non-significant, these trends might achieve significance in a larger cohort or with extended follow-up.

### Dissociation between biomarkers and ventilatory parameters

Although RIPC significantly reduced CC16, IL-6, and MDA levels, no parallel improvements were observed in OR, RI, A-aDO_2_, or a/A ratio. This dissociation may arise because biomarkers are more sensitive indicators of early epithelial and inflammatory injury, whereas gas exchange parameters require more pronounced or prolonged dysfunction to change. In addition, the present cohort had relatively preserved baseline lung function, and the 30-minute post-OLV observation window may have been too short for functional effects to emerge. Similar findings have been reported, where reductions in the inflammatory biomarkers after RIPC or OLV were not accompanied by immediate improvements in oxygenation or respiratory mechanics, although improvements in oxygenation (PaO_2_/FiO_2_) were observed at later postoperative time points (2‒24 h).^24^^,^^25^ These results suggest that RIPC may confer protection at a cellular and molecular level, with functional benefits becoming evident only over longer follow-up or in higher-risk patients. No post-hoc correlation analyses were performed between biomarker changes and oxygenation indices, as the trial was not powered for exploratory association testing. Future studies with larger sample sizes may help clarify whether molecular improvements translate into measurable physiological benefits.

### Clinical implications and novelty

One strength of the present study is the application of five RIPC cycles, in contrast to the more commonly used three cycles, allowing for potential enhancement of protective efficacy. Additionally, these findings support the use of non-invasive, low-cost, and easily applicable RIPC as a feasible adjunct to lung-protective strategies during thoracic surgery.

### Limitations

Several limitations warrant mention. First, the modest overall sample size limited the power to detect clinical differences in functional outcomes.

In particular, the biomarker with the study design and prior literature. Although statistically significant differences in CC16, IL-6 and MDA were observed, the relatively small biomarker sample size means that these findings should be interpreted with caution, and confirmation in larger, adequately powered studies is required.

Second, the authors restricted observation to 30-minutes post-surgery, precluding analysis of delayed or sustained protective effects. Third, this study did not explore other potential mechanisms, such as TNF-α, HIF-1α, or anti-inflammatory cytokines like IL-10, which might have elucidated additional pathways through which RIPC mediates lung protection. Finally, the single-center design may limit the generalizability of these findings to other institutions with different perioperative protocols and patient populations.

## Conclusion

In summary, this single-blind, randomized controlled clinical trial demonstrates that remote ischemic preconditioning may attenuate lung epithelial injury, inflammatory cytokine production, and oxidative stress in patients undergoing thoracoscopic lobectomy under OLV. These effects were reflected in differences in plasma CC16, IL-6, and MDA levels in a predefined biomarker sub-sample. Although no significant differences were observed in oxygenation indices or clinical outcomes, the biomarker findings support a potential protective effect of RIPC. As a non-invasive, convenient, and cost-effective intervention, RIPC shows potential for perioperative lung protection. However, these preliminary findings require confirmation in larger, adequately powered multicenter studies to establish its clinical efficacy and elucidate underlying mechanisms.

## Declaration of generative AI and AI-assisted technologies in the writing process

During the preparation of this work, the author(s) used ChatGPT (OpenAI) to assist with drafting and refining sections of the manuscript, primarily to improve the clarity and quality of language expression. After using this tool, the author(s) reviewed and edited the content as needed and take(s) full responsibility for the content of the publication.

## Data availability

All data are available within the text.

## CRediT authorship contribution statement

**Wenfu Zhang:** Investigation, Data curation, Writing – original draft. **Mingwang Zeng:** Investigation, Data curation. **Chao Yang:** Investigation, Visualization. **Lijun Yang:** Investigation, Visualization. **Juan Yang:** Visualization, Formal analysis, Software. **Yi Wang:** Formal analysis, Software. **Haiyu Xie:** Investigation. **Lifeng Wang:** Investigation. **Maolin Zhong:** Supervision, Resources. **Fuzhou Hua:** Supervision, Methodology. **Weidong Liang:** Conceptualization, Writing – original draft, Writing – review & editing, Resources, Project administration, Funding acquisition.

## Declaration of competing interest

The authors declare that they have no known competing financial interests or personal relationships that could have appeared to influence the work reported in this paper.
